# The SoftHand Pro: Functional evaluation of a novel, flexible, and robust myoelectric prosthesis

**DOI:** 10.1371/journal.pone.0205653

**Published:** 2018-10-15

**Authors:** Sasha Blue Godfrey, Kristin D. Zhao, Amanda Theuer, Manuel G. Catalano, Matteo Bianchi, Ryan Breighner, Divya Bhaskaran, Ryan Lennon, Giorgio Grioli, Marco Santello, Antonio Bicchi, Karen Andrews

**Affiliations:** 1 Soft Robotics for Human Collaboration and Rehabilitation Lab, Department of Advanced Robotics, Istituto Italiano di Tecnologia, Genoa, GE, Italy; 2 Assistive and Restorative Technology Laboratory, Rehabilitation Medicine Research Center, Mayo Clinic, Rochester, MN, United States of America; 3 Department of Physical Medicine and Rehabilitation, Mayo Clinic, Rochester, MN, United States of America; 4 Centro di Ricerca E. Piaggio, University of Pisa, Pisa, PI, Italy; 5 Department of Health Sciences Research, Mayo Clinic, Rochester, MN, United States of America; 6 Neural Control of Movement Laboratory, School of Biological and Health Systems Engineering, Arizona State University, Tempe, AZ, United States of America; 7 School of Biological and Health Systems Engineering, Arizona State University, Tempe, AZ, United States of America; University of Illinois at Urbana-Champaign, UNITED STATES

## Abstract

Roughly one quarter of active upper limb prosthetic technology is rejected by the user, and user surveys have identified key areas requiring improvement: function, comfort, cost, durability, and appearance. Here we present the first systematic, clinical assessment of a novel prosthetic hand, the SoftHand Pro (SHP), in participants with transradial amputation and age-matched, limb-intact participants. The SHP is a robust and functional prosthetic hand that minimizes cost and weight using an underactuated design with a single motor. Participants with limb loss were evaluated on functional clinical measures before and after a 6–8 hour training period with the SHP as well as with their own prosthesis; limb-intact participants were tested only before and after SHP training. Participants with limb loss also evaluated their own prosthesis and the SHP (following training) using subjective questionnaires. Both objective and subjective results were positive and illuminated the strengths and weaknesses of the SHP. In particular, results pre-training show the SHP is easy to use, and significant improvement in the Activities Measure for Upper Limb Amputees in both groups following a 6–8 hour training highlights the ease of learning the unique features of the SHP (median improvement: 4.71 and 3.26 and p = 0.009 and 0.036 for limb loss and limb-intact groups, respectively). Further, we found no difference in performance compared to participant’s own commercial devices in several clinical measures and found performance surpassing these devices on two functional tasks, buttoning a shirt and using a cell phone, suggesting a functional prosthetic design. Finally, improvements are needed in the SHP design and/or training in light of poor results in small object manipulation. Taken together, these results show the promise of the SHP, a flexible and adaptive prosthetic hand, and pave a path forward to ensuring higher functionality in future.

## Introduction

The human hand is important for many activities of daily living (ADL), including self-feeding, tool use, and recreation, and thus loss of the upper extremity has a large impact on functional independence, psychological well-being, and overall quality of life [1]. While exact global statistics are unknown, the WHO estimates 16% of amputations affect the upper limb [2]. A combination of technical complexity and limited market size hinder upper limb prosthetic advances that leap forward in fits and starts, often motivated by increased visibility and awareness, such as that caused by war or medical problems [3].

Myoelectric prostheses have been around since the 1960s and transform residual muscle signals into commands for a powered, electric prosthetic terminal device [3]. Despite advances in technology since their debut, upper extremity prosthetic function and satisfaction remain low: the adult rejection rate for myoelectric upper limb prostheses is estimated at 23% [4]. Most often, these prostheses resemble a human hand, but have an internal tri-digit structure that closes in a C-shape for power or pinch grasp. Less common are myoelectric greifers and similar technologies that offer higher grip force and are more amenable to manual labor but are not anthropomorphic. Both types of devices allow simple, voluntary control in both open and close directions and perform a single, rigid grasp. Over the last decade, a new generation of anthropomorphic myoelectric hands debuted [5], offering persons with limb loss multiple grasp postures with the goal of enabling greater function and convenience while improving aesthetics. These, however, are heavier [5] and more expensive, in terms of both initial cost and maintenance. Further, the control complexity of such a device demands a higher cognitive burden on the part of the user to fully access the widened feature set [6] and may thus result in a prosthesis that is not utilized to its capacity.

Body-powered prostheses offer an alternative for users who do not desire and/or are unable to use myoelectric prostheses. These devices are typically not anthropomorphic. The most common all-purpose terminal device is a hook [4, 7], which is very robust and can be very functional when used as a tool; however, not all users are able to become sufficiently proficient in its use. Other activity-specific terminal devices are often custom-made for the individual user and must be switched out as needed. For individuals with transradial limb loss, body-powered devices are typically controlled by a figure-of-nine harness through movement of the contralateral shoulder [1]. This type of control allows easy activation and provides a measure of sensory feedback of aperture and grip force [8]; however, it can also cause shoulder pain or injury and motivate device abandonment [1]. Although these devices are quite different from their myoelectric counterparts, their rejection rate is quite similar (26%) [4], and while myoelectric and body-powered prostheses each exhibit specific strengths and weaknesses, neither provide an overall advantage over the other [9].

Beyond the rejection rate of specific prosthesis types, non-wear, or choosing not to wear a prosthesis as opposed to rejecting a specific type of device, and passive use of upper limb prostheses (regardless of type) are estimated at 20 and 27%, respectively, indicating a high level of dissatisfaction with available technology [4, 10]. The two most important design criteria for both body-powered and myoelectric hands, as ranked by prosthesis users, are function and comfort [11]. These are followed by cost, durability, and appearance, in differing order of importance. Individuals with limb loss thus face a gap in available prosthetic technology: an easy-to-use, lightweight, robust, and low maintenance anthropomorphic prosthetic hand.

Research efforts are taking a multi-faceted approach to improving upper limb prosthetic technology. These include exploring alternative control methods, such as pattern recognition to allow the user to more naturally control multiple degrees of freedom [12] and automating slip prevention and compliant grasping [13]; crafting new invasive techniques such as targeted muscle reinnervation [14] and implantable myoelectric sensors [15] to improve control signal strength and resolution; and designing new sophisticated hands. While a review of all of these approaches is out of the scope of this work, a brief summary of research efforts in prosthetic hand development is relevant and warranted. Many groups are focused on producing more human-like hands that offer multiple discrete postures, often using multiple motors [5]. The UNB Hand, for example, features precision, tripod, cylindrical, and lateral grips and uses a combination of pattern recognition and conventional control methods [16]. The prosthetic hand presented in [17] has four degrees of actuation driving eight grasps or postures (including open-hand) using 2-site myocontrol. With the aim of providing a truly lightweight hand, the Lightweight Delft Cylinder Hand was designed as a body-powered device that uses hydraulic power to lessen the burden on the driving shoulder [18]. While this is not an exhaustive list, it illustrates the inherent trade-off in prosthetics between the user needs described above: increasing the mechanical complexity to improve function often requires control schemes that are not fully robust to real-world conditions, or place a larger burden on the user than conventional systems, while using body-power reduces this control complexity at the cost of shifting at least some of the physical burden of actuation to the user.

In this paper, we present results of clinical testing of a new type of prosthetic hand, the SoftHand Pro (SHP), which brings versatile, human-like movements to an easily-controlled and robust prosthetic hand to address the gaps outlined above. Its design is based on the innovative approach of “soft synergies” used in the University of Pisa/IIT Robotic SoftHand [19, 20] designed for robotics applications. The approach, which capitalizes on the combination of recent scientific understanding of human hand synergies [21] and novel soft robotics technologies, has introduced a new paradigm in prosthetic design. The SHP has all of the degrees of freedom of a natural human hand, including articulating DIP (distal interphalangeal) joints, which are often rigid in prosthetic hands; however, since it is driven by a single motor, the control burden of the user is minimized. The SHP can be used to grasp a wide variety of common objects and is resistant to large impacts. Previously, the original SoftHand under myoelectric control had been tested only on limb-intact volunteers with a forearm adapter (e.g. [22, 23] with the aim of exploring prosthetic applications. Many novel prosthetic devices are first tested on limb-intact volunteers to avoid over-burdening the small population with limb-loss and to improve the rate of iteration in research. However, the extent of the utility of such studies remains an open question. The pilot study presented in this work is the first clinical evaluation of this novel prosthetic prototype, the SoftHand Pro, in participants with upper extremity limb loss and age- and hand dominance-matched, limb-intact participants. This study aimed to compare the functionality of the SoftHand Pro to participants’ own prosthetic devices, examine intuitiveness and ease of learning of the SHP, and also provide a first comparison of the results of a group of participants with limb-loss with those of limb-intact participants in a controlled setting.

## Materials and methods

### Study design

A pilot group of 9 participants with transradial amputations (8 males, 1 female; mean age: 51 years ± 18.9 years, Table 1) were tested at the Mayo Clinic in Rochester, MN using the SHP. Nine limb-intact, age- and hand dominance-matched limb-intact participants (7 females and 2 males) were also tested wearing the SHP via a forearm adapter. Limb-intact participants were age-matched to within plus or minus two years of a participant with transradial amputation. Hand dominance of limb-intact participants was matched to dominance prior to amputation in participants with limb loss; limb-intact participants then wore the SHP on the amputated side of their matched participant. The study was approved by the Mayo Clinic Institutional Review Board (IRB) on 10/13/2014, and all participants provided written consent prior to participating in the study. All images included in this work are of participants who gave their explicit, written consent to use their (unidentifiable) images. Participants with limb loss completed a battery of clinical evaluations and questionnaires with their own prosthesis on the first day of the study. All participants were trained on use of the SHP by an occupational therapist and completed the same battery of tests before and after training. A detailed description of the protocol follows.

**Table 1:** Demographics of participants with limb loss.

Participant	Age at time of testing	Time since Amputation (at time of study)	Side Amputated	Previous hand dominance	Gender	Own Prosthesis *	Alternate Prosthesis
1	67	8	R	R	M	Multigrasp MP	
2	56	33	R	R	M	BP hook	
3	72	1	R	R	M	Multigrasp MP	MP hook
4	35	6.5	R	R	M	BP hook	
5	27	14	R	L	M	BP hook	
6	45	18.5	L	R	M	BP hand	BP hook
7	77	3.5	R	R	M	BP hook	
8	53	53	L	R	F	Tridigit MP	
9	27	3	L	R	M	BP hook	Multigrasp MP

* MP indicates myoelectric prosthesis; BP indicates body-powered

### SoftHand Pro

**Fig 1:**
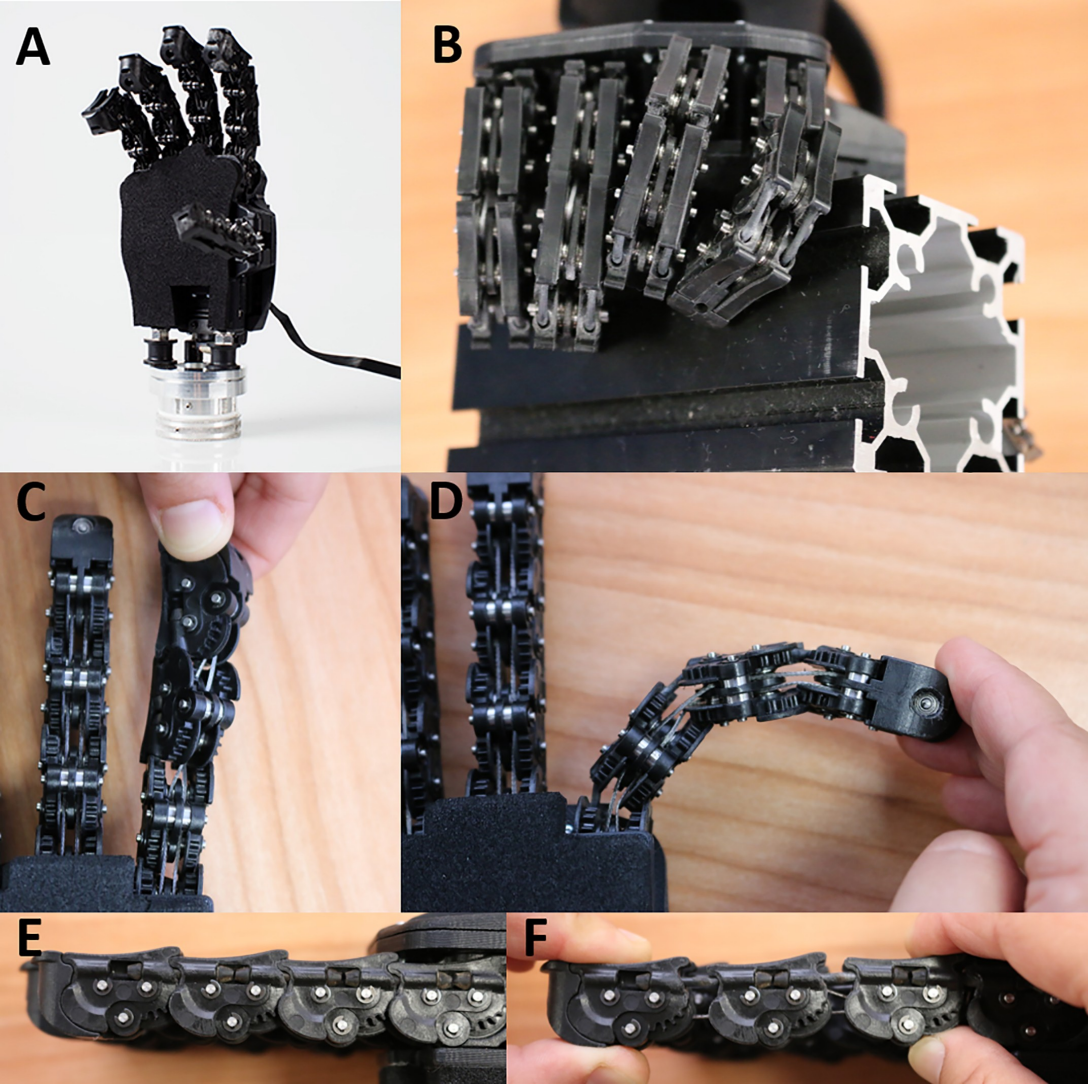
The SoftHand Pro. A: The SoftHand Pro shown with wrist interface. B: The SHP grasping a large square tube taking advantage of flexible joint design. Bottom two rows: Demonstrating SHP twisting (C), bending (D), and disarticulating (E, F) capabilities.

As mentioned above, the SoftHand Pro (SHP) draws inspiration from the 19-degree of freedom Pisa/IIT SoftHand [20]. In brief, the SHP, like its predecessor, is an anthropomorphic prosthetic hand that follows the first kinematic hand movement synergy, as defined by principal component analysis [21], to coordinate all movements of the fingers and thumb using a single motor. The joints of the fingers are floating joints brought into proximity axially by elastic bands on the dorsal side, rather than rigidly fixed together allowing flexion/extension but not separation, as can be found in commercial prostheses. This non-rigid coupling provides two of the key features of the SoftHand and SoftHand Pro that, to our knowledge, are not found in other devices. First, the synergistic pattern the hand follows acts as a kind of “baseline trajectory” in the absence of interaction forces but allows for deviations in their presence to enable a conformal grasp, due not only to the aforementioned non-rigid coupling but also the SHP’s differential drive. Second, the joints are able to hyperextend, twist, or even dislocate temporarily and then return to position automatically. This ability was designed to increase the robustness of the SH and SHP, preventing damage in the event of unexpected impacts or collisions. Further, this robustness can be particularly useful in taking advantage of object properties and features of the surrounding environment, together the environmental constraints, to enable new grasp patterns. Fig 1 shows the SHP on its own and grasping a large (6 cm) square tube as well as close-ups of some of the less-conventional joint features (hyperextension not shown). Note: the SHP is used with a glove to improve grasping but is shown here without one to illustrate various features more clearly. The grasp image shows the proximal interphalangeal joint of the index finger and the metacarpal phalangeal joint of the middle finger out of alignment with respect to more proximal segments; the misalignment results from the flexible joint design and enables a conformal grasp. For more detail on the mechanical implementation and demonstration of these features, please see Catalano et al. 2014 and Bonilla et al. 2014 [20, 24].

The SHP used in the experiments described in this paper (Fig 1) approximates the size of a large male hand, weighing 520 g, with a length (from base of hand to middle finger tip) of 200 mm and a width of 90 mm at the palm; note that smaller versions of the SHP, in sizes that would better fit an average female or even a child are being developed. The electronics and motor are housed on the dorsal side of the hand. To better interface with a prosthetic socket, a quick disconnect style wrist component was developed that allowed manual pronation and supination. Further, to allow passive wrist extension, the wrist was flexibly connected to the SHP using compact rubber dampers. As the hand pushes against a surface, for example the fingers and/or palm against a table in grasping or against an armrest to assist standing, the wrist passively and temporarily bends into extension, up to approximately 60°. Note, the wrist extension is activated exclusively through the application of external forces. Further, while active wrist flexion may be a useful feature in future, passive wrist flexion via the compact rubber dampers was mechanically blocked to improve function. The SHP provides 76 N of force in power grasp and 20 N in pinch and is capable of a lifting force of 400 N. Finally, the SHP is myoelectrically controlled using two commercial surface electromyography (EMG) electrodes (Otto Bock, Germany). Because the SHP has only one motor, advanced myoelectric controllers, such as pattern recognition or dexterous control, are not required. Three different myoelectric control modes were used in this study, all of which allow for proportional control of the SHP and hold position when the muscles are at rest. Integral Control was based on the difference between the extensor and flexor (open and close) signals allowing participants to rapidly change direction and fine-tune the input to the device. For participants with difficulty controlling co-contraction, First Come, First Served (FCFS) and an advanced version of the FCFS were available: the former takes into consideration the first signal to go above a minimum threshold and is controlled by only that signal until it drops below threshold. The latter requires both signals to drop below threshold before allowing the user to potentially switch direction.

### Study protocol

**Fig 2:**
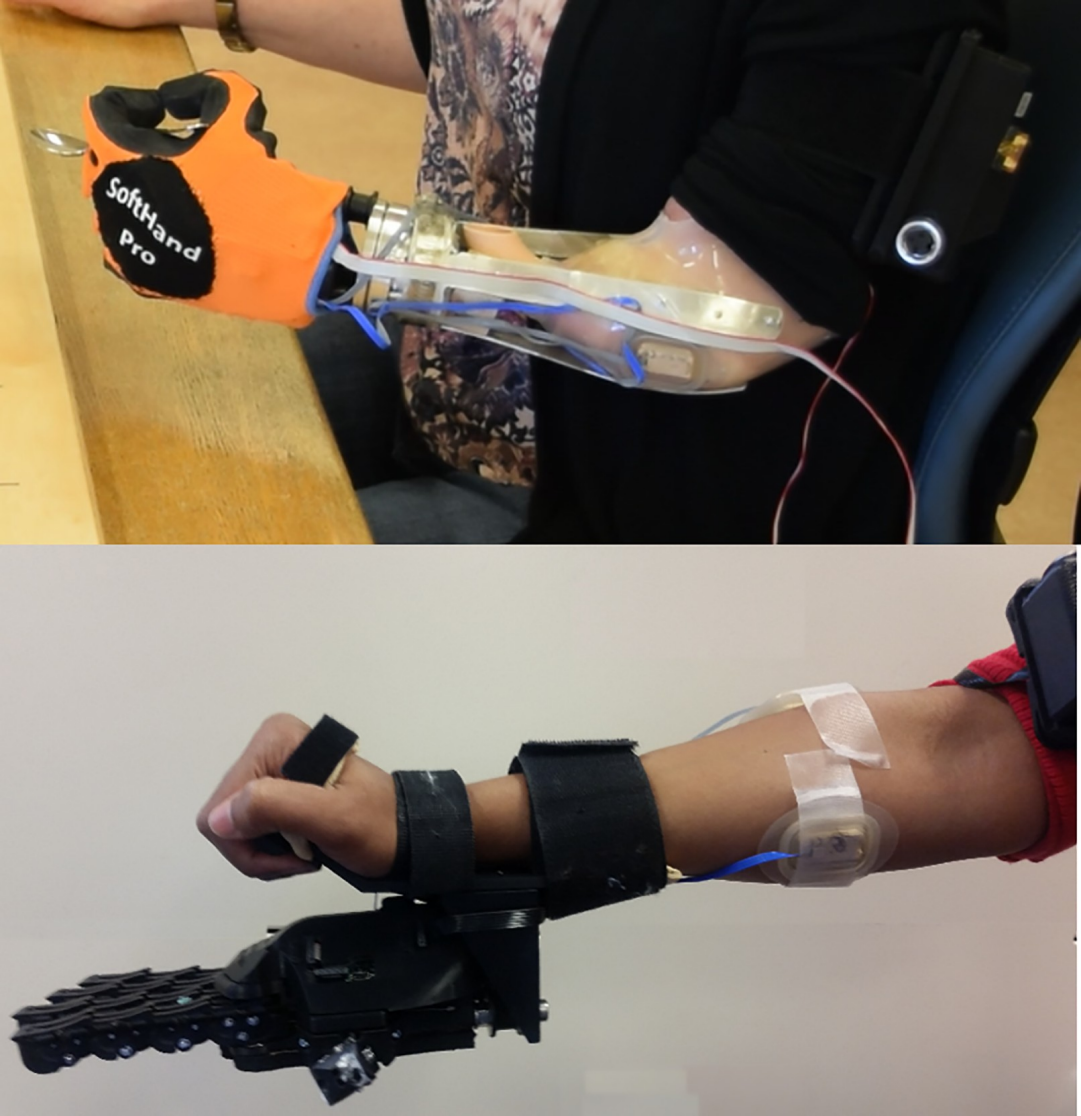
Participants wearing the SoftHand Pro. Top: The SHP attached to a myoelectric socket used by a participant with limb loss. Bottom: The SHP attached to a forearm adapter used by limb-intact participants.

After enrollment, participants with limb loss had a custom prosthetic socket built by the study prosthetist (Fig 2 top); participants with intact limbs wore the SHP below their natural hand, using a forearm adapter to don the SHP, as shown in Fig 2 bottom. Participants with limb loss completed a battery of clinical assessments with their own prosthesis on the first day of the study; participants were tested without undergoing any study intervention (i.e.: occupational therapy training) related to their own prosthesis to faithfully record their functional level with their preferred prosthesis. All participants completed these assessments using the SHP before and after training with an occupational therapist. Participants with limb loss also responded to subjective surveys/questionnaires regarding their own prosthesis and the SHP following training. Surveys were omitted from the SHP pre-training assessment as familiarity with the prosthesis was needed to provide an informed response to survey questions. Similarly, surveys were omitted entirely from the battery of testing for intact-limb participants as most questions were not relevant, and it would have been unreasonable for them to extrapolate from in-study exposure of prosthetic technology to the real-world impact of such technology on their daily life.

**Fig 3:**
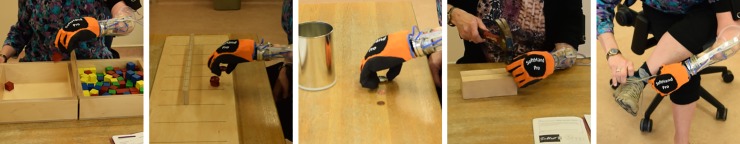
Clinical evaluation of prosthesis. Examples of participants completing the clinical measures. From left to right, Box and Blocks, the Jebsen Taylor Test of Hand Function (stacking checkers, moving small, common objects), and the AM-ULA (hammering a nail, shoe tying).

Systematic collection and analysis of outcomes data are challenging for studies of persons with upper-limb amputation. The Upper Limb Prosthetic Outcome Measures (ULPOM) Working Group aimed to develop a tool kit of validated measures addressing each major domain of the International Classification of Functioning, Disability, and Health [25]. Following recommendations from the ULPOM, we used the Activities Measure for Upper Limb Amputees (AM-ULA) [26], an 18-item measure of activity performance for adults with upper-limb amputations. (Note: we removed the liquid pouring task due to IRB restrictions.) The AM-ULA considers task completion, speed, movement quality, skill of prosthetic use, and independence in its rating system. This measure has excellent internal consistency, good interrater reliability, test-retest reliability, and demonstrated known-group and convergent validity. We used the Box and Blocks (B&B) [27] test, consisting of moving 1 inch blocks from a box, over a partition, and into another box, to quantify gross manual dexterity and speed. Additionally, the Jebsen Taylor Test of Hand Function (JTHF, Jebsen) [28], which tests 7 simulated ADLs from writing to feeding to moving large and small objects, to evaluate ADL performance in terms of time to completion. Both the B&B and Jebsen are clinical tests that are typically used to quantify impaired hand function. Fig 3 provides examples of participants with upper limb loss completing the clinical assessments. We also included two surveys in the assessment, the Canadian Occupational Performance Measure (COPM) [29] and the short version of the Disabilities of the Hand, Arm, and Shoulder questionnaire (QuickDASH) [30], to qualitatively represent the participant’s performance in everyday life with the prosthesis and satisfaction with that performance. In the COPM, users are asked to choose up to five ADLs that are personally important and then rate their performance and satisfaction on those tasks, whereas the QuickDASH asks responders to rate how much arm impairment impacts a list of 6 ADLs, social activities, and work and further asks questions related to pain severity and impact. Note that the two surveys were omitted from the SHP pre-training assessment.

Participants that had limited or no recent experience with myoelectrically-controlled prostheses were given myoelectric training (MT) before testing with the SoftHand Pro. MT focused on teaching basic myoelectric operation, rather than specific features of the SHP, in order to minimize the difference in myoelectric control ability between those subjects that did not have previous experience with myoelectric control and those that did. Prior to the pre-testing, all participants were given a brief (roughly 30 min) period to familiarize themselves with the SHP and to become comfortable controlling the SoftHand Pro as opposed to their typical prosthesis. Participants were able to choose between the three control modes described above based on personal preference.

**Fig 4:**
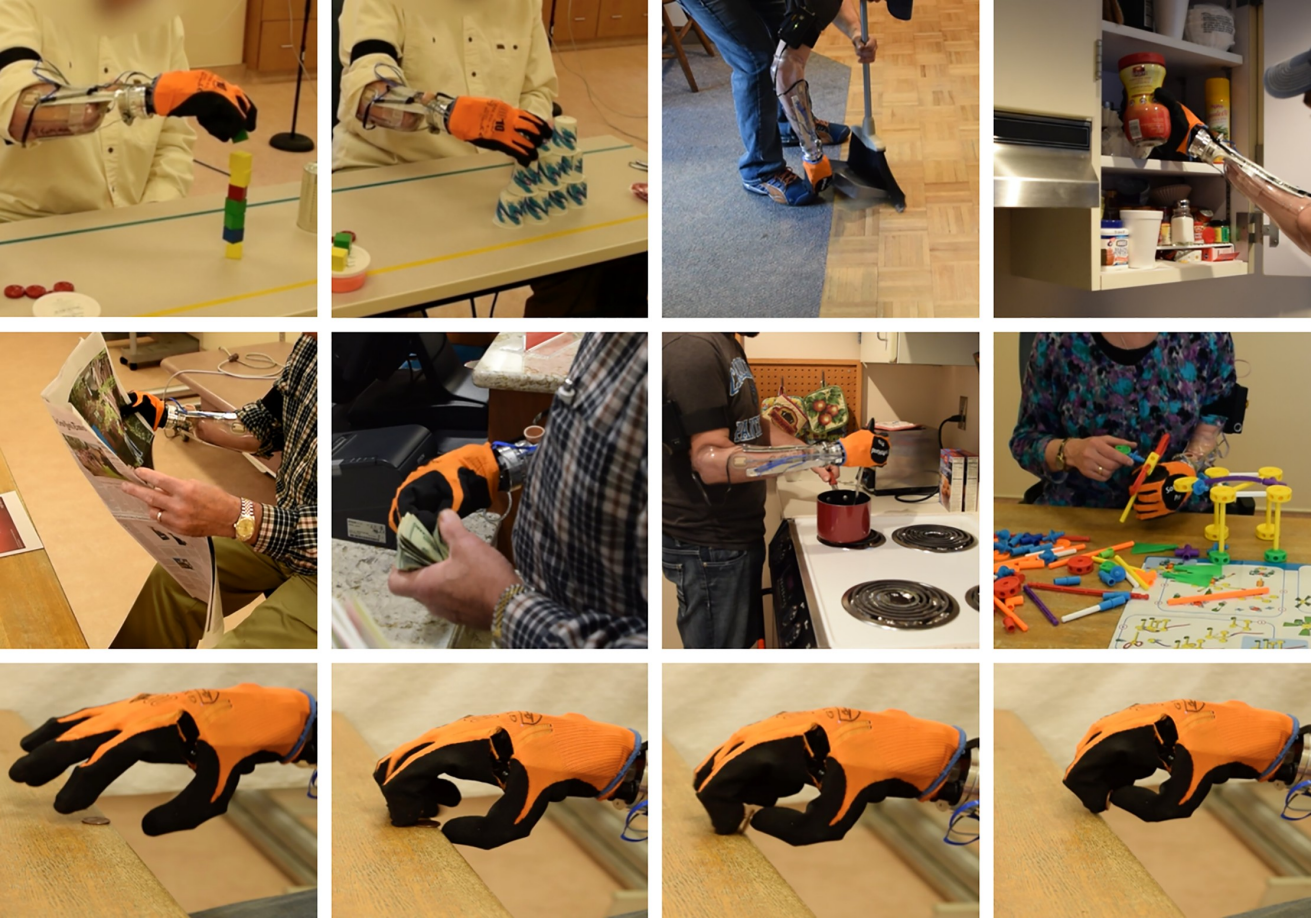
SoftHand Pro training. An example progression through training starting from simple, repeated grasp tasks (top row, left two) to real-world tasks exploring the work space (top row, right two) to coordinated bimanual tasks, including hobby and leisure activities (middle row). The bottom row shows a participant practicing using environmental constraints to pick up a coin (US penny) from a table. The movement (left to right) starts with pre-grasping, proceeds to blocking the thumb against the table edge, closing the fingers to meet the table and coin, sliding the coin to the edge while bringing the thumb up to meet it, and ends with grasp completion.

Participants then trained with an occupational therapist on use of the SoftHand Pro for approximately six to eight hours over two-days. This training progressed through basic open-close control of the hand, grasping and moving objects of different shapes and sizes, and bimanual and collaborative ADLs. Once the participant had mastered basic use of the SoftHand Pro, training emphasized the SHP’s unique ability to deform by using environmental constraints to affect the shape of the hand’s closure. Fig 4 shows examples of various training activities, demonstrating this progression. More specifically, training began focusing on controlling open and closing movements of the hand, learning to modulate the aperture of the hand and control the force. Examples of training exercises included grasping fragile (plastic or paper) cups or single cubes and progressing to stacking cups or cubes into a pyramid. Basic one-handed and bimanual tasks were then targeted: for example, simulating a buffet line by carrying objects in the prosthetic hand while manipulating objects with the other hand; exploring the workspace by picking items off the floor or a shelf; building small toy models. In the later stages of training, participants played board or card games with study staff, encouraging natural use of the prosthesis in a social setting, practiced ADL tasks in a therapy apartment, and practiced with hobby equipment they had brought from home (e.g. golf clubs, tools, etc). The timing of the different phases of training was not regimented but rather followed the order given above and progressed to more and more difficult tasks based on the study therapist’s judgement. This study design was chosen to tailor the training to each participant, allowing them to progress at their own pace ensuring that all participants had a solid foundation but avoided boredom and fatigue by varying tasks and including breaks as needed. Immediately after training, participants were retested with the full assessment as described above. Table 2 below provides a summary of how the outcome measures were scored; further information can be found in the cited references.

**Table 2:** Outcome measure overview.

Full Name of Test	Test Short Name	Scoring Method	Score Range	Unimpaired Score
Disabilities of the Arm, Shoulder, and Hand (Quick version)	QuickDASH	self-rated from 1 (no limitation) to 5 (unable), then scaled from 0–100	0–100	0
Canadian Occupational Performance Measure	COPM	self-rated on scale of 1 (poor) to 10 (excellent)	1–10	10
Box and Blocks	B&B	number of blocks in 1 minute	0 –N/A	N/A
Activities Measure for Upper Limb Amputees	AM-ULA	rated by OT from 0 (unable) to 4 (excellent) on performance, then averaged and multiplied by 10	0–40	40
Jebsen Taylor Test of Hand Function	Jebsen/JTHF	timed by OT per task. (We imposed a 120 second limit to limit frustration and fatigue)	0–120	N/A; faster is less impaired

### Data analysis

Variables are summarized with percentiles (median, 25^th^, 75^th^ percentile) unless otherwise noted; we chose to use the median rather than the mean, because with a small sample, outliers and skewed distributions could be particularly influential on the mean. Three time points were considered and compared: testing with participants’ own prostheses, with the SHP pre-training, and with the SHP post-training. Differences between participants’ own prostheses and the post-training SHP performance, as well as before and after SHP training were calculated. The Wilcoxon signed rank test was used to test for significant differences between paired measures (pre- versus post-training, and SHP post-training versus own prosthesis). Participants had 120 seconds to complete each Jebsen sub-task. If the task took longer than 120 seconds, it was considered a “fail”. For purposes of analysis, “fail” trials are valued at 120 seconds, and the calculated difference between a failed attempt and a successful attempt was also set at 120 seconds. For example, if a participant was not able to complete a sub-task in the SHP pre-training testing but was able in the post-training testing, the calculated difference upon which the statistical analysis was performed was set to 120 s, regardless of the time recorded on the successful trial. Non-parametric statistics such as median and the signed-rank test are invariant to changes in values as long as the ordering of the values remains the same, thus our results are unaffected by the choice of 120 as the fail value as any value of 120 or larger would give identical results. P-values less than 0.05 were declared statistically significant and were used to identify substantial differences. No adjustments for multiple hypothesis tests were done. While we acknowledge that our p-values would lose significance if adjusted for multiple comparisons, we do not believe this is the most appropriate treatment for this data [31]. Due to the modest sample size of this pilot study, we have not performed statistical analyses on group subsets, for example, where this kind of adjustment is often indicated. Further, all measures are reported with means, medians, and p-values in supplementary tables.

## Results

As mentioned in the Data Analysis section above, two primary analyses were performed: the first to compare performance with the SHP to that with participants’ own prostheses, and the second to look at the effect of the SHP training on SHP performance. To facilitate interpretation of the results, please refer to Table 2 in the Materials & Methods section that summarizes the outcome measures used. Data are presented as the median difference (MD) of the two time-points indicated along with the interquartile range (IQR, 25^th^ to 75^th^ percentile range) and, where appropriate, p value.

### Primary analyses

**Fig 5:**
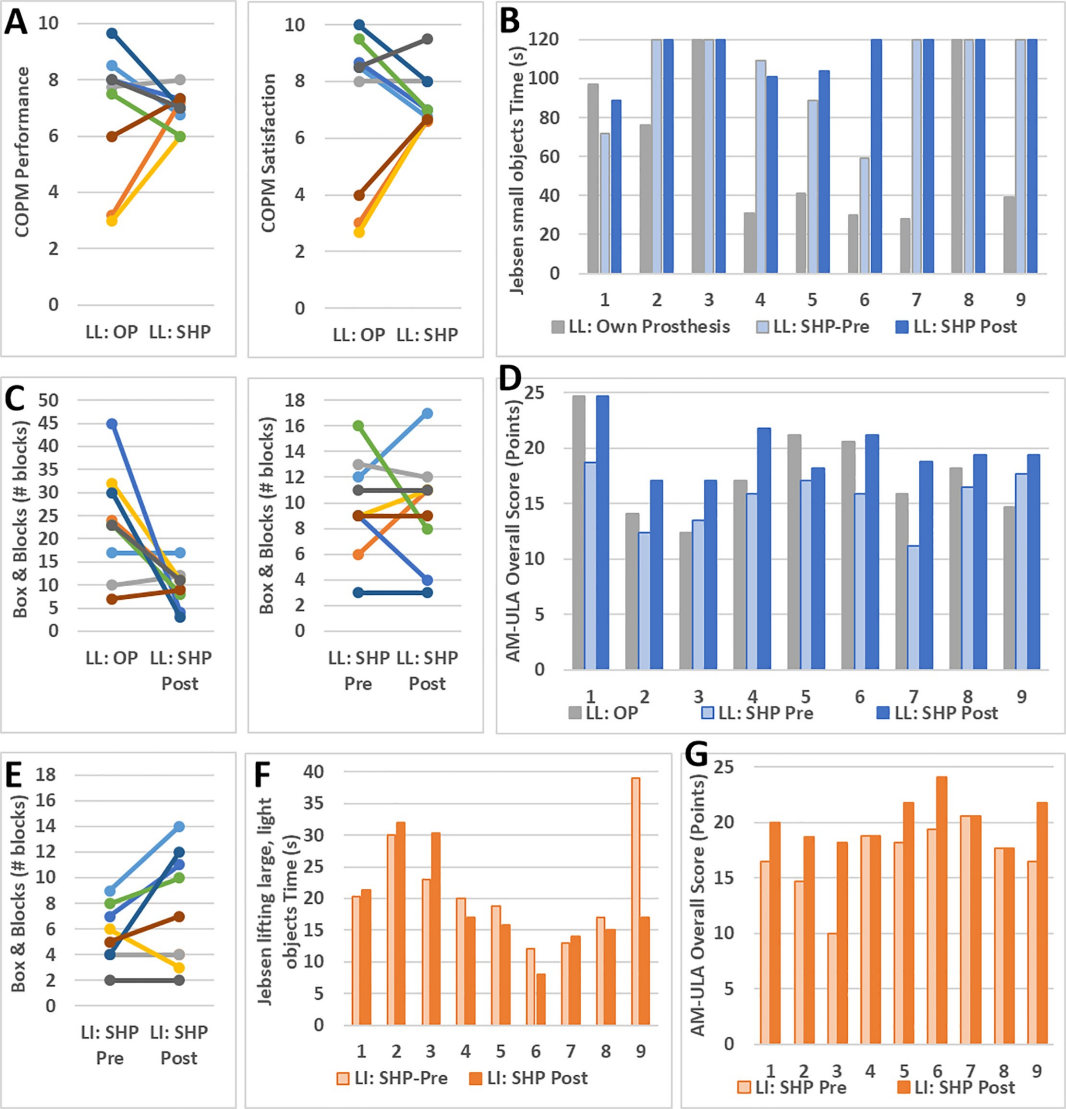
Primary analysis results. Comparison of SHP (post-training) to participants’ own prostheses in the COPM (top left) and all three time points for Jebsen “moving small, common objects” (top right), B&B (bottom left) and AM-ULA (bottom right). B&B, Jebsen “lifting large, light objects,” and AM-ULA results of limb-intact participants (LI) shown in the bottom row. Participants with limb loss are denoted as “LL” and own prosthesis results are denoted “OP.” Matched LL and LI participants are denoted using the same color (B&B) or number (AM-ULA and Jebsen).

Results from participants with limb loss with the SHP post-training were compared with the results from their own prosthesis. No significant differences were found between participants’ own prostheses and SHP post-training on the COPM (Fig 5A) and QuickDASH questionnaires. Participants performed significantly better with their own prosthesis compared to the SHP on the B&B (Fig 5C; MD: 13 blocks; IQR: 0–21 blocks; p = 0.042) and on three Jebsen subtasks: lifting small, common objects (Fig 5B), stacking checkers, and lifting large, heavy objects (MD, IQR: 70, 43–103; 22, 16–95; 9, 3–12 seconds and p = 0.021, 0.044, and 0.018, respectively). In contrast, they performed significantly better with the SHP compared to their own prosthesis on AM-ULA subtasks: buttoning shirt and using a cell phone (MD, IQR: 1, 0–1; 2, 1–2 points and p = 0.026 and 0.027, respectively). As can be seen in Fig 5D, the overall AM-ULA results, though they did not reach the level of significance (MD, IQR: 2.94, 0.59–4.70 p = 0.080), were very positive with 7 out of 9 participants with limb loss improving in overall score with the SHP compared to their own prosthesis, 3 of whom exceeded the minimum detectable change. Of the two remaining participants, one performed equally well with both prostheses, and was the highest performer of the group, whereas the other performed worse with the SHP compared to their own prosthesis.

The effect of training was examined in both participants with limb loss and age matched limb-intact participants. Both groups improved significantly with training on the overall AM-ULA (Fig 5D and 5G) score: 4.71 median increase in points (IQR: 2.94–5.88 points, p = 0.009) for participants with limb loss and 3.26 median increase (IQR: 0–4.71, p = 0.036) for limb-intact participants. Looking at the breakdown of the individual AM-ULA tasks, participants with limb loss improved significantly on the spoon and phone tasks (p = 0.026 and 0.048, respectively; median improvement of 1 point and IQR 0–1 for each task). Limb-intact participants showed significant improvement on fork, towel, and shelf tasks (p = 0.011, 0.037, and 0.037, respectively; median improvement of 1 point and IQR 0–1 on each task). B&B did not show a training effect in either group. None of the Jebsen sub-tasks were significantly different post- compared to pre-training in the limb-intact group. In participants with limb loss, there was a median improvement of 9 seconds (IQR: 3–21 seconds, p = 0.018) in moving large, light objects.

### Secondary analyses

**Fig 6:**
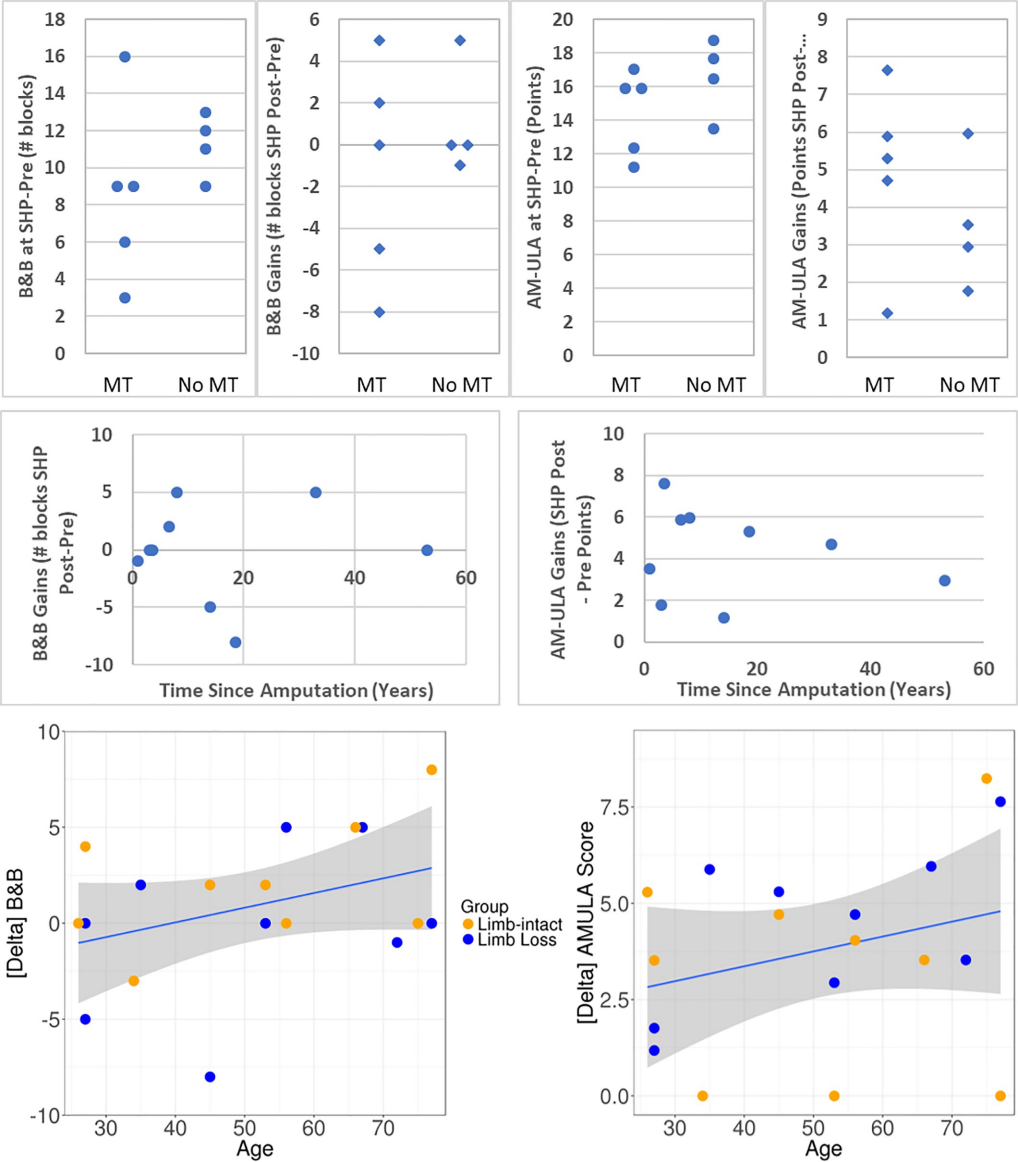
Secondary analysis results. Top row: B&B and AM-ULA (left two and right two graphs, respectively) comparison of participants with and without additional myoelectric training (MT), in terms of SHP Pre-testing scores and gains (SHP Post-testing scores minus pre-testing). Middle and bottom rows: B&B and AM-ULA (left and right, respectively) gains plotted against time since amputation (middle row) and participant age (bottom). Linear regression line and confidence limits (shaded region) are also shown in bottom row.

The participants with amputation in this study had varying degrees of experience using myoelectric prostheses. To further understand the results of this study, we separated the participants with amputation into two groups by level of myoelectric experience: those who participated in MT (additional myoelectric training) prior to pre-testing with the SoftHand Pro (n = 5), and those who already had sufficient experience prior to pre-testing. These two groups coincide almost perfectly with those whose own prosthesis was body-powered and those whose own prosthesis was myoelectric. (Participant 9 is an exception: he brought a body-powered hook prosthesis as his main prosthesis but also had extensive practice with his alternate prosthesis, a microprocessor myoelectric hand (iLimb, Touch Bionics, UK)). Though the two groups were too small to compare with statistical analysis, there was no clear difference between the two groups in terms of starting performance (points in pre-testing) or training gains (as measured by difference between post- and pre-testing values) in B&B, AM-ULA, and Jebsen tests, although the participants who had previous myoelectric experience appear to perform more similarly within group than their counterparts. Fig 6 top row shows the pre-testing values and training gains for the B&B and AM-ULA tests. Similarly, no differences were evident in the survey (QuickDASH and COPM) or AM-ULA or most Jebsen results related to whether the participant’s own prosthesis was body-powered or myoelectric. Body-powered (BP) prostheses, however, appeared to perform better on B&B and the Jebsen “moving small, common objects” task than the three myoelectric prostheses. In B&B, the median for the whole group was 23 blocks; participants with BP prostheses moved between 23 and 45 blocks whereas the three participants with myoelectric prostheses (MP) moved 7, 10, and 17 blocks each. S9 who had both a BP and myoelectric prosthesis, however, obtained similar results with both (23 and 22, respectively).Of the participants with MPs, only one of the three completed the small object task (in 97 s); the five participants with body-powered hooks, however, took between 28 and 76 seconds to complete the small object task, with all but one participant finishing in 41 s or less.

Finally, participants spanned a wide age range (27 to 77 years), which may have influenced results as some clinical measures show age-related correlations (eg: decreased performance in B&B and JTHF with increasing age, [32, 33]). To explore this aspect, we plotted age against change in outcome measure and calculated the correlation. While none of the correlations was statistically significant due to small sample size, we found that in 20 of 26 correlations, there was a tendency for older participants to show greater improvement following training. Two examples of this finding are shown in Fig 6 (bottom row). However, when score was plotted against time since amputation rather than age (Fig 6, middle row), no such tendencies were evident, suggesting, in combination with the above observations related to type of prosthesis (myoelectric or body-powered) or amount of myoelectric training, that the tendencies observed are likely related to participant age rather than other factors.

## Discussion

The SoftHand Pro is an anthropomorphic hand with 19 joints but a single actuator, so digits close simultaneously according to a synergistic pattern of movement derived from intact human hand movements. Further, the hand is adaptive and flexible, thereby allowing it to conform to a wide variety of object shapes and sizes. These two features, following a synergistic pattern and adapting flexibly to environmental constraints, are not found in commercially-available devices, to the best of the authors’ knowledge. This study evaluated the novel SHP in a clinical laboratory environment via two primary comparisons: comparing results obtained with the SHP following 6–8 hours of occupational therapy against SHP results pre-training and against the participants’ own prosthetic device. The former comparison was performed both with participants with and without limb-loss, while the latter, by necessity, only with participants with limb-loss. In SHP pre-testing, both experienced and naïve users performed at a reasonable level following a minimal (up to 30 minute) familiarization period, suggesting ease of use of a prosthetic device with a non-rigid (and thus variable) closure pattern. Further, statistically significant improvements were made in the relatively brief (6–8 hours) training, as shown by the significant gains in the AM-ULA that surpassed or approached the minimum detectable change for both participants with limb loss and age-matched, limb-intact participants, respectively. These results indicate that control of the unique aspects of the SHP can be gained even with limited exposure. One of the Jebsen subtasks (lifting small, common objects) showed a significant decrease in performance (measured as time to task completion) and other tasks similarly showed slight (non-significant) decreases or remained flat with training. The study occupational therapist noted that movements were often more controlled and precise in post-testing, likely accounting for some of the paradoxical decrease in performance (as measured by speed) with training. Results from the AM-ULA, which rate completion of ADLs using more criteria than simply speed, hint at this improved quality of performance with practice. A few participants noted they were more nervous (had test anxiety) in post-testing compared to pre-testing. Participants were reassured to simply try their best and not worry about their score, but this anxiety likely decreased performance on some tasks. Additionally, post-testing was performed at the end of the second day of training with the SHP, whereas pre-testing was performed at the start of the first day, thus fatigue potentially played a role in post-testing performance. Modifying the study design in future work should limit the effects of this confounding factor.

While it would be reasonable to hypothesize that results would necessarily improve following training, this was not the case in all of our outcome measures. There is limited literature looking at the effects of training on use of prosthetic technology [34] and variety in study design hinders comparison between works (ie: different outcome measures used, case study design, hours of training, level of amputation [34, 35, 36, 37]). In Resnik and Borgia, 2016, for example, 39 individuals with amputations (of which 12 were transradial) participated in extensive training (> 20 hours) on the DEKA arm. Outcome measures in common between the study in this work and Resnik and Borgia were the B&B, AM-ULA, and the JTHF, comprised of 7 subtests. It is worth noting that the baseline testing in Resnik and Borgia took place after a virtual reality training (approximately 2 hours) and a brief familiarization period. Looking at the subset of subjects with transradial amputations and the outcome measures included also in the present work, after ten 10 hours of training 6 out of 9 outcome measures had a positive effect size, although 8 had confidence intervals that crossed zero. Following an additional 10 hours of training, there was an increase in effect size on 5 of the 9 outcome measures. Notably, Jesbsen subtasks “lifting small, common objects” and “stacking checkers” had small, negative effect sizes following 10 hours of training, which became more negative (although still small, -0.12 and -0.26, respectively) following the full training. Dromerick et al. 2008 presented a pediatric case study of a 15 y.o. male with transhumeral (left) and scapular disarticulation (right) amputations. Training occurred over an 8 week period, totaling roughly 19 hours with testing before, during (after roughly 11 hours), and after training; outcome measures in common with the present study were the B&B and JTHF. As in the study presented here as well as Resnik and Borgia, not all outcome measures improved following training: the subject showed a decrease in performance in three Jebsen subtasks (writing, lifting small, common objects, and stacking checkers) following 11 hours of training, all of which improved to better-than-baseline with additional training. The other two studies cited (Lake 1997 and Bouwsema et al. 2008) had outcome measures that did not overlap with the present study; the former used a modified version of the University of New Brunswick (UNB) test while the latter focused on ADL-based tasks. Summarizing the training results, limb-intact and limb loss groups showed similar gains overall, although there appeared to be a tendency for wider variation in performance in the limb-intact group, probably owing to being naïve prosthesis users. Further, we found the Jebsen resistant to training effects, as had other groups, while the AM-ULA showed more consistent improvement. It is important, however, to note that both the Resnik and Borgia and Dromerick et al. studies found positive effects of training in the B&B. Neither of our groups exhibited such an effect, suggesting that our training methods and/or duration may need to be adjusted in concert with the planned mechanical improvements, elaborated on below.

Overall, the SHP performed well compared to participants’ own prostheses, especially considering the limited exposure and training with the SHP and the potential fatigue effects mentioned above. Particularly noteworthy are the results of the AM-ULA that showed an increase in performance with the SHP compared to participants’ own prostheses in 7 out of 9 participants. These results suggest that the SHP is a highly functional prosthesis for use in real-world tasks. The SHP underperformed with respect to participants’ prostheses on the Box and Blocks test and in the “lifting small, common objects” and “stacking checkers” subtasks of the Jebsen test; these negative results may be attributable to several factors. Five of the participants used body-powered hooks as their typical prosthesis. These terminal devices are particularly adept at precision grasping tasks (and thus are often favored as work prostheses). Similarly, it is interesting to note that in the B&B and “lifting small, common objects” Jebsen subtask, the three participants with myoelectric hands had the lowest performance within the “own prosthesis” group (see details in Results). In addition to the fact that body-powered prostheses, in particular hooks, may provide an advantage in certain precision tasks, the SHP’s flexible and adaptive grasp, in which all digits move together, may require further training to master manipulation of small objects, in particular pre-positioning and using the surrounding environment. Design changes are also being implemented to further facilitate small object grasping with the SHP in the future. Subjective results, as seen in the COPM, showed that participants performed well with the SHP and were satisfied with their performance (upper half of COPM range, median of 7 points for both measures). As can be seen in the COPM plots in the results, participants displayed a wide range of performance and satisfaction with their own prostheses (range 3–10); these ratings tended to be less variable for the SHP, with the participants who had the most extreme views of their own prosthesis showing larger changes in rating than those with more temperate ratings. Taken as a whole, the qualitative results seem to suggest the SHP was found to be functional and satisfying, despite limited exposure to the SHP and the variety of prostheses used by study participants in daily life. While not assayed systematically, we noted participants with limb loss using myoelectric prostheses tended to have a more timid or gentle approach to handling objects, perhaps due to a perception of fragility with these devices. The SHP’s engineered flexibility, conversely, encouraged and sometimes necessitated new approaches to grasping problems, which could potentially open new avenues for functionality not originally imagined when designing the hand. In future studies, it would be interesting to query this directly in a subjective questionnaire to distinguish whether participants perceive themselves to be using different strategies with the SHP, if those strategies arise out of need or possibility, and if participants would be more gentle with the SHP were it their everyday prosthesis (i.e. that they are responsible to maintain).

The heterogeneity of the participant group was qualitatively examined. The lack of apparent differences in training effect suggests that the MT provided for BP users was an effective method to minimize the effects of differences in myoelectric control experience. Further, the type of prosthesis each participant used did not seem to influence results in comparison to SHP post-training results, with the potential exception of B&B in which BP prostheses generally performed better. It is possible, though, that the age of the participants played a role in the study results. There was an apparent tendency for the training effect to increase with participant age. While we do not have sufficient statistical power to rigorously test this result, participant age should indeed be taken into account in future study design. Younger participants may be more amenable to new technology after a short familiarization period, and thus have less room for improvement with training relative to older participants. Interestingly, the time since amputation did not appear to play a role in study results, suggesting that all participants had a reasonable base of experience with prostheses.

### Additional considerations and study limitations

As mentioned above in the Materials and Methods section, the AM-ULA has been developed and subsequently tested for use in individuals with amputation and thus has been shown to be a reliable and valid outcome measure for this population. The B&B and JTHF, however, were first developed for use in other populations. Indeed, they are shown to be reliable and valid in individuals following stroke or traumatic brain injury or with multiple sclerosis [38, 39]. In individuals with amputation, however, there is limited data validating these measures. A recent paper by Resnik and Borgia [40] found the B&B to have excellent reliability, while the various subtasks of the JTHF showed acceptable to good reliability with the exception of one subtask, which showed excellent reliability. These results, however, should be interpreted with caution: Resnik and Borgia evaluated two alternative methods for scoring the JTHF, counting number of items moved within a two-minute time limit and calculating items moved per second. The latter methodology proved more reliable in 5 out of 7 subtasks. However, the study presented herein used the more standard methodology, adding only a time-limit to each subtask (2 minutes) and thus rating both incomplete attempts and successes outside of that range as failures. Further work is needed to fully validate this methodology in this population.

The participants with limb loss represent a very diverse group with ages ranging from 27 to 77 years and time since amputation ranging from 1 to 33 years (plus one participant with congenital limb loss). They varied in side amputated (6 right and 3 left) and whether the hand lost was previously dominant (5 participants had their dominant hand amputated). Further, participants had varying amounts of experience with myoelectric terminal devices, although efforts were made in-study to bring all participants to a reasonable baseline level of myoelectric control before pre-testing with the SHP. As discussed above, apart from participant age, these factors do not appear to have influenced the results but cannot be fully excluded without further study. Although this heterogeneity can be seen as a limitation of this study, it is also a strength as the results are valid across the vast diversity of the limb loss community rather than in a specific, selected sub-group. The limb-intact group, though age- and hand dominance-matched, was not matched for gender, which may confound limb-intact group results. Additionally, participants had only 6–8 hours of training with the SHP. Future studies will include sending the SHP home to increase overall exposure to the device and better test its performance in real-world, everyday tasks. While we included simulated real-world tasks, for example practicing in a therapy apartment, actual home use over a longer period would potentially also impact subjective measures, as participants would be better able to rate the functionality of the prosthesis and their satisfaction with its use in everyday life. Finally, it would be meaningful in the future to examine reaching trajectories and compensatory motions used with the SHP related to other prostheses: given the involvement of the contralateral shoulder in controlling BP prostheses, one might expect noticeable differences between these two conditions. It is also possible that the adaptive nature of the SHP would result in different approach strategies than those seen when using MPs.

## Conclusions

This work presents the first clinical testing of the SoftHand Pro with participants with limb loss. The results show that, as an adaptive, anthropomorphic hand, the SHP is easy to use and highly functional both for individuals experienced in myoelectric prosthetic control and novices. The study showed that the SHP performed extremely well on functional tasks (AM-ULA) but also revealed features of the SHP that can be improved in the future (small object manipulation). The novel design of the SHP represents a true departure from currently available technology and has been seen, in this study, to be a viable path forward for a functional and well-accepted prosthetic hand.

## Supporting information

S1 Table SoftHand Pro versus own prosthesis. The table below presents summary statistics for participants with limb loss comparing performance with the SoftHand Pro and their own prosthesis. The p-value is from a signed rank test to test if the median change (delta) is significantly different from zero.
(DOCX)

S2 Table Training effect (Delta) in participants with limb loss. The table below presents summary statistics for participants with limb loss before and after training with the SoftHand Pro. The p-value is from a signed rank test to test if the median change is significantly different from zero.
(DOCX)

S3 Table Training effect (Delta) in limb-intact participants. The table below presents summary statistics for limb-intact participants before and after training with the SoftHand Pro. The p-value is from a signed rank test to test if the median change is significantly different from zero. Note: The Jebsen “writing” sub-task was not performed in limb-intact participants.
(DOCX)
