# Supplementary material for: The SoftHand Pro: Functional evaluation of a novel, flexible, and robust myoelectric prosthesis
Source: PLoS One. 2018 Oct 15;13(10):e0205653. doi: 10.1371/journal.pone.0205653 (PMC6188862; doi:10.1371/journal.pone.0205653)
Supplement: S1 Table — The table below presents summary statistics for participants with limb loss comparing performance with the SoftHand Pro and their own prosthesis. The p-value is from a signed rank test to test if the median change (delta) is significantly different from zero. (DOCX) [file pone.0205653.s001.docx]

**S1 Table. SoftHand Pro versus own prosthesis.** The table below presents summary statistics for participants with limb loss comparing performance with the SoftHand Pro and their own prosthesis. The p-value is from a signed rank test to test if the median change (delta) is significantly different from zero.

| **Measure** | **Mean** | **SD** | **Median** | **25th %ile** | **75th %ile** | **P-value** |
| --- | --- | --- | --- | --- | --- | --- |
| Quick DASH : Overall |  |  |  |  |  | 0.575 |
| - Delta | 2.02 | 10.32 | 2.27 | -2.27 | 9.09 |  |
| - Own Prosthesis | 19.19 | 12.45 | 20.45 | 9.09 | 27.27 |  |
| - Post | 21.20 | 7.95 | 22.73 | 18.18 | 27.27 |  |
| COPM : Performance |  |  |  |  |  | 1.000 |
| - Delta | 0.11 | 2.25 | -0.67 | -1.50 | 1.33 |  |
| - Own Prosthesis | 6.85 | 2.33 | 7.75 | 6.00 | 8.00 |  |
| - Post | 6.96 | 0.64 | 7.00 | 6.75 | 7.33 |  |
| COPM : Satisfaction |  |  |  |  |  | 0.624 |
| - Delta | 0.37 | 2.55 | 0.00 | -1.75 | 2.67 |  |
| - Own Prosthesis | 6.98 | 2.90 | 8.50 | 4.00 | 8.67 |  |
| - Post | 7.35 | 0.97 | 7.00 | 6.67 | 8.00 |  |
| B&B : Score |  |  |  |  |  | 0.042 |
| - Delta | -13.89 | 14.41 | -13.00 | -21.00 | 0.00 |  |
| - Own Prosthesis | 23.44 | 11.59 | 23.00 | 17.00 | 30.00 |  |
| - Post | 9.56 | 4.25 | 11.00 | 8.00 | 11.00 |  |
| AMULA : Score |  |  |  |  |  | 0.080 |
| - Delta | 2.09 | 2.61 | 2.94 | 0.59 | 4.70 |  |
| - Own Prosthesis | 17.65 | 3.94 | 17.05 | 14.71 | 20.59 |  |
| - Post | 19.74 | 2.46 | 19.41 | 18.24 | 21.18 |  |
| Jebsen : Writing |  |  |  |  |  | 0.673 |
| - Delta | 0.00 | 24.33 | 0.00 | -18.00 | 1.00 |  |
| - Own Prosthesis | 42.89 | 20.61 | 45.00 | 30.00 | 55.00 |  |
| - Post | 42.89 | 28.30 | 31.00 | 29.00 | 47.00 |  |
| Jebsen : Simulated page turning |  |  |  |  |  | 0.553 |
| - Delta | 4.44 | 27.00 | 4.00 | -12.00 | 22.00 |  |
| - Own Prosthesis | 38.78 | 16.40 | 42.00 | 22.00 | 53.00 |  |
| - Post | 43.22 | 27.03 | 36.00 | 26.00 | 42.00 |  |
| Jebsen : Lifting small, common objects |  |  |  |  |  | 0.035 |
| - Delta | 48.00 | 40.71 | 63.00 | 0.00 | 81.00 |  |
| - Own Prosthesis | 64.67 | 39.04 | 41.00 | 31.00 | 97.00 |  |
| - Post | 112.67 | 11.69 | 120.00 | 104.00 | 120.00 |  |
| Jebsen : Simulated feeding |  |  |  |  |  | 0.138 |
| - Delta | 6.44 | 15.52 | 3.00 | 1.00 | 15.00 |  |
| - Own Prosthesis | 15.11 | 8.07 | 13.00 | 11.00 | 15.00 |  |
| - Post | 21.56 | 12.71 | 15.00 | 13.00 | 30.00 |  |
| Jebsen : Stacking checkers |  |  |  |  |  | 0.044 |
| - Delta | 36.44 | 44.17 | 22.00 | 16.00 | 73.00 |  |
| - Own Prosthesis | 33.33 | 18.53 | 28.00 | 20.00 | 47.00 |  |
| - Post | 69.78 | 41.26 | 67.00 | 29.00 | 120.00 |  |
| Jebsen : Lifting large, light objects |  |  |  |  |  | 0.476 |
| - Delta | 3.11 | 8.61 | 2.00 | -4.00 | 7.00 |  |
| - Own Prosthesis | 13.67 | 5.48 | 14.00 | 10.00 | 14.00 |  |
| - Post | 16.78 | 7.41 | 13.00 | 11.00 | 24.00 |  |
| Jebsen : Lifting large, heavy objects |  |  |  |  |  | 0.018 |
| - Delta | 7.78 | 6.92 | 9.00 | 3.00 | 12.00 |  |
| - Own Prosthesis | 12.78 | 4.18 | 13.00 | 10.00 | 17.00 |  |
| - Post | 20.56 | 4.98 | 20.00 | 17.00 | 25.00 |  |
| AMULA comp : comb |  |  |  |  |  | 0.129 |
| - Delta | 0.44 | 0.73 | 1.00 | 0.00 | 1.00 |  |
| - Own Prosthesis | 1.78 | 0.67 | 2.00 | 1.00 | 2.00 |  |
| - Post | 2.22 | 0.67 | 2.00 | 2.00 | 3.00 |  |
| AMULA comp : t-shirt on |  |  |  |  |  | 0.408 |
| - Delta | -0.33 | 1.12 | 0.00 | -1.00 | 0.00 |  |
| - Own Prosthesis | 1.11 | 1.27 | 1.00 | 0.00 | 2.00 |  |
| - Post | 0.78 | 0.67 | 1.00 | 0.00 | 1.00 |  |
| AMULA comp : t-shirt off |  |  |  |  |  | 0.710 |
| - Delta | 0.22 | 1.09 | 0.00 | 0.00 | 1.00 |  |
| - Own Prosthesis | 0.67 | 1.12 | 0.00 | 0.00 | 1.00 |  |
| - Post | 0.89 | 1.17 | 0.00 | 0.00 | 2.00 |  |
| AMULA comp : button shirt |  |  |  |  |  | 0.026 |
| - Delta | 0.78 | 0.67 | 1.00 | 0.00 | 1.00 |  |
| - Own Prosthesis | 0.56 | 0.53 | 1.00 | 0.00 | 1.00 |  |
| - Post | 1.33 | 0.71 | 1.00 | 1.00 | 2.00 |  |
| AMULA comp : zipper |  |  |  |  |  | 0.072 |
| - Delta | 0.44 | 0.53 | 0.00 | 0.00 | 1.00 |  |
| - Own Prosthesis | 1.67 | 0.71 | 2.00 | 1.00 | 2.00 |  |
| - Post | 2.11 | 0.60 | 2.00 | 2.00 | 2.00 |  |
| AMULA comp : sock |  |  |  |  |  | 0.850 |
| - Delta | 0.11 | 0.93 | 0.00 | 0.00 | 1.00 |  |
| - Own Prosthesis | 1.89 | 1.17 | 2.00 | 1.00 | 3.00 |  |
| - Post | 2.00 | 0.87 | 2.00 | 1.00 | 3.00 |  |
| AMULA comp : shoe |  |  |  |  |  | 0.168 |
| - Delta | -0.56 | 1.01 | 0.00 | -1.00 | 0.00 |  |
| - Own Prosthesis | 2.56 | 0.73 | 3.00 | 2.00 | 3.00 |  |
| - Post | 2.00 | 0.71 | 2.00 | 2.00 | 2.00 |  |
| AMULA comp : cup |  |  |  |  |  | 0.484 |
| - Delta | 0.22 | 0.83 | 0.00 | 0.00 | 1.00 |  |
| - Own Prosthesis | 2.00 | 0.71 | 2.00 | 2.00 | 2.00 |  |
| - Post | 2.22 | 0.83 | 2.00 | 2.00 | 3.00 |  |
| AMULA comp : fork |  |  |  |  |  | 0.484 |
| - Delta | 0.22 | 0.83 | 0.00 | 0.00 | 1.00 |  |
| - Own Prosthesis | 1.78 | 0.67 | 2.00 | 1.00 | 2.00 |  |
| - Post | 2.00 | 0.50 | 2.00 | 2.00 | 2.00 |  |
| AMULA comp : spoon |  |  |  |  |  | 0.203 |
| - Delta | 0.44 | 0.88 | 0.00 | 0.00 | 1.00 |  |
| - Own Prosthesis | 1.67 | 0.50 | 2.00 | 1.00 | 2.00 |  |
| - Post | 2.11 | 0.60 | 2.00 | 2.00 | 2.00 |  |
| AMULA comp : writing |  |  |  |  |  | 0.773 |
| - Delta | -0.11 | 0.60 | 0.00 | 0.00 | 0.00 |  |
| - Own Prosthesis | 2.33 | 0.50 | 2.00 | 2.00 | 3.00 |  |
| - Post | 2.22 | 0.44 | 2.00 | 2.00 | 2.00 |  |
| AMULA comp : cutting |  |  |  |  |  | 0.424 |
| - Delta | -0.22 | 0.67 | 0.00 | -1.00 | 0.00 |  |
| - Own Prosthesis | 2.67 | 0.50 | 3.00 | 2.00 | 3.00 |  |
| - Post | 2.44 | 0.53 | 2.00 | 2.00 | 3.00 |  |
| AMULA comp : doorknob |  |  |  |  |  | 0.129 |
| - Delta | 0.67 | 1.12 | 0.00 | 0.00 | 2.00 |  |
| - Own Prosthesis | 1.22 | 1.09 | 1.00 | 0.00 | 2.00 |  |
| - Post | 1.89 | 0.33 | 2.00 | 2.00 | 2.00 |  |
| AMULA comp : phone |  |  |  |  |  | 0.027 |
| - Delta | 1.33 | 1.22 | 2.00 | 1.00 | 2.00 |  |
| - Own Prosthesis | 1.11 | 1.05 | 1.00 | 0.00 | 2.00 |  |
| - Post | 2.44 | 0.53 | 2.00 | 2.00 | 3.00 |  |
| AMULA comp : hammer |  |  |  |  |  | 0.766 |
| - Delta | -0.11 | 0.78 | 0.00 | -1.00 | 0.00 |  |
| - Own Prosthesis | 2.00 | 0.71 | 2.00 | 2.00 | 2.00 |  |
| - Post | 1.89 | 0.60 | 2.00 | 2.00 | 2.00 |  |
| AMULA comp : towel |  |  |  |  |  | 0.484 |
| - Delta | -0.22 | 0.83 | 0.00 | -1.00 | 0.00 |  |
| - Own Prosthesis | 2.56 | 0.73 | 3.00 | 2.00 | 3.00 |  |
| - Post | 2.33 | 0.71 | 2.00 | 2.00 | 3.00 |  |
| AMULA comp : overhead shelf |  |  |  |  |  | 0.484 |
| - Delta | 0.22 | 0.83 | 0.00 | 0.00 | 1.00 |  |
| - Own Prosthesis | 2.44 | 0.73 | 3.00 | 2.00 | 3.00 |  |
| - Post | 2.67 | 0.50 | 3.00 | 2.00 | 3.00 |  |
