# Supplementary material for: The SoftHand Pro: Functional evaluation of a novel, flexible, and robust myoelectric prosthesis
Source: PLoS One. 2018 Oct 15;13(10):e0205653. doi: 10.1371/journal.pone.0205653 (PMC6188862; doi:10.1371/journal.pone.0205653)
Supplement: S2 Table — The table below presents summary statistics for participants with limb loss before and after training with the SoftHand Pro. The p-value is from a signed rank test to test if the median change is significantly different from zero. (DOCX) [file pone.0205653.s002.docx]

**S2 Table. Training effect (Delta) in participants with limb loss.** The table below presents summary statistics for participants with limb loss before and after training with the SoftHand Pro. The p-value is from a signed rank test to test if the median change is significantly different from zero.

| **Measure** | **Mean** | **SD** | **Median** | **25th %ile** | **75th %ile** | **P-value** |
| --- | --- | --- | --- | --- | --- | --- |
| B&B : Score |  |  |  |  |  | 1.000 |
| - Delta | -0.22 | 4.24 | 0.00 | -1.00 | 2.00 |  |
| - Pre | 9.78 | 3.83 | 9.00 | 9.00 | 12.00 |  |
| - Post | 9.56 | 4.25 | 11.00 | 8.00 | 11.00 |  |
| AMULA : Score |  |  |  |  |  | 0.009 |
| - Delta | 4.32 | 2.13 | 4.71 | 2.94 | 5.88 |  |
| - Pre | 15.42 | 2.53 | 15.88 | 13.52 | 17.06 |  |
| - Post | 19.74 | 2.46 | 19.41 | 18.24 | 21.18 |  |
| Jebsen : Writing |  |  |  |  |  | 0.362 |
| - Delta | -8.12 | 33.50 | -2.50 | -18.25 | 0.25 |  |
| - Pre | 54.38 | 27.16 | 50.00 | 33.00 | 62.75 |  |
| - Post | 42.89 | 28.30 | 31.00 | 29.00 | 47.00 |  |
| Jebsen : Simulated page turning |  |  |  |  |  | 0.363 |
| - Delta | -13.22 | 29.80 | -2.00 | -20.00 | 3.00 |  |
| - Pre | 56.44 | 40.73 | 41.00 | 22.00 | 72.00 |  |
| - Post | 43.22 | 27.03 | 36.00 | 26.00 | 42.00 |  |
| Jebsen : Lifting small, common objects |  |  |  |  |  | 0.201 |
| - Delta | 9.44 | 20.91 | 0.00 | 0.00 | 15.00 |  |
| - Pre | 103.22 | 23.91 | 120.00 | 89.00 | 120.00 |  |
| - Post | 112.67 | 11.69 | 120.00 | 104.00 | 120.00 |  |
| Jebsen : Simulated feeding |  |  |  |  |  | 0.529 |
| - Delta | -16.44 | 41.11 | 0.00 | -9.00 | 2.00 |  |
| - Pre | 38.00 | 41.13 | 20.00 | 11.00 | 35.00 |  |
| - Post | 21.56 | 12.71 | 15.00 | 13.00 | 30.00 |  |
| Jebsen : Stacking checkers |  |  |  |  |  | 0.446 |
| - Delta | -13.78 | 46.39 | 0.00 | -53.00 | 3.00 |  |
| - Pre | 83.56 | 38.17 | 78.00 | 56.00 | 120.00 |  |
| - Post | 69.78 | 41.26 | 67.00 | 29.00 | 120.00 |  |
| Jebsen : Lifting large, light objects |  |  |  |  |  | 0.018 |
| - Delta | -18.33 | 25.82 | -9.00 | -21.00 | -3.00 |  |
| - Pre | 35.11 | 27.99 | 24.00 | 18.00 | 47.00 |  |
| - Post | 16.78 | 7.41 | 13.00 | 11.00 | 24.00 |  |
| Jebsen : Lifting large, heavy objects |  |  |  |  |  | 0.483 |
| - Delta | -5.67 | 15.30 | -2.00 | -7.00 | 2.00 |  |
| - Pre | 26.22 | 16.81 | 22.00 | 15.00 | 32.00 |  |
| - Post | 20.56 | 4.98 | 20.00 | 17.00 | 25.00 |  |
| AMULA comp : comb |  |  |  |  |  | 0.129 |
| - Delta | 0.44 | 0.73 | 1.00 | 0.00 | 1.00 |  |
| - Pre | 1.78 | 0.67 | 2.00 | 1.00 | 2.00 |  |
| - Post | 2.22 | 0.67 | 2.00 | 2.00 | 3.00 |  |
| AMULA comp : t-shirt on |  |  |  |  |  | 0.572 |
| - Delta | 0.22 | 0.97 | 0.00 | 0.00 | 1.00 |  |
| - Pre | 0.56 | 0.88 | 0.00 | 0.00 | 1.00 |  |
| - Post | 0.78 | 0.67 | 1.00 | 0.00 | 1.00 |  |
| AMULA comp : t-shirt off |  |  |  |  |  | 0.203 |
| - Delta | 0.44 | 0.88 | 0.00 | 0.00 | 1.00 |  |
| - Pre | 0.44 | 0.73 | 0.00 | 0.00 | 1.00 |  |
| - Post | 0.89 | 1.17 | 0.00 | 0.00 | 2.00 |  |
| AMULA comp : button shirt |  |  |  |  |  | 0.346 |
| - Delta | 0.22 | 0.44 | 0.00 | 0.00 | 0.00 |  |
| - Pre | 1.11 | 0.60 | 1.00 | 1.00 | 1.00 |  |
| - Post | 1.33 | 0.71 | 1.00 | 1.00 | 2.00 |  |
| AMULA comp : zipper |  |  |  |  |  | 0.174 |
| - Delta | 0.44 | 0.73 | 0.00 | 0.00 | 1.00 |  |
| - Pre | 1.67 | 0.71 | 2.00 | 1.00 | 2.00 |  |
| - Post | 2.11 | 0.60 | 2.00 | 2.00 | 2.00 |  |
| AMULA comp : sock |  |  |  |  |  | 0.089 |
| - Delta | 0.56 | 0.73 | 0.00 | 0.00 | 1.00 |  |
| - Pre | 1.44 | 0.53 | 1.00 | 1.00 | 2.00 |  |
| - Post | 2.00 | 0.87 | 2.00 | 1.00 | 3.00 |  |
| AMULA comp : shoe |  |  |  |  |  | 0.120 |
| - Delta | 0.56 | 0.88 | 1.00 | 0.00 | 1.00 |  |
| - Pre | 1.44 | 0.53 | 1.00 | 1.00 | 2.00 |  |
| - Post | 2.00 | 0.71 | 2.00 | 2.00 | 2.00 |  |
| AMULA comp : cup |  |  |  |  |  | 0.129 |
| - Delta | 0.50 | 0.76 | 1.00 | 0.00 | 1.00 |  |
| - Pre | 1.62 | 0.52 | 2.00 | 1.00 | 2.00 |  |
| - Post | 2.22 | 0.83 | 2.00 | 2.00 | 3.00 |  |
| AMULA comp : fork |  |  |  |  |  | 0.089 |
| - Delta | 0.56 | 0.73 | 0.00 | 0.00 | 1.00 |  |
| - Pre | 1.44 | 0.53 | 1.00 | 1.00 | 2.00 |  |
| - Post | 2.00 | 0.50 | 2.00 | 2.00 | 2.00 |  |
| AMULA comp : spoon |  |  |  |  |  | 0.026 |
| - Delta | 0.78 | 0.67 | 1.00 | 0.00 | 1.00 |  |
| - Pre | 1.33 | 0.50 | 1.00 | 1.00 | 2.00 |  |
| - Post | 2.11 | 0.60 | 2.00 | 2.00 | 2.00 |  |
| AMULA comp : writing |  |  |  |  |  | 0.149 |
| - Delta | 0.33 | 0.50 | 0.00 | 0.00 | 1.00 |  |
| - Pre | 1.89 | 0.60 | 2.00 | 2.00 | 2.00 |  |
| - Post | 2.22 | 0.44 | 2.00 | 2.00 | 2.00 |  |
| AMULA comp : cutting |  |  |  |  |  | 0.072 |
| - Delta | 0.44 | 0.53 | 0.00 | 0.00 | 1.00 |  |
| - Pre | 2.00 | 0.50 | 2.00 | 2.00 | 2.00 |  |
| - Post | 2.44 | 0.53 | 2.00 | 2.00 | 3.00 |  |
| AMULA comp : doorknob |  |  |  |  |  | 0.129 |
| - Delta | 0.44 | 0.73 | 1.00 | 0.00 | 1.00 |  |
| - Pre | 1.44 | 0.73 | 1.00 | 1.00 | 2.00 |  |
| - Post | 1.89 | 0.33 | 2.00 | 2.00 | 2.00 |  |
| AMULA comp : phone |  |  |  |  |  | 0.048 |
| - Delta | 0.67 | 0.71 | 1.00 | 0.00 | 1.00 |  |
| - Pre | 1.78 | 0.44 | 2.00 | 2.00 | 2.00 |  |
| - Post | 2.44 | 0.53 | 2.00 | 2.00 | 3.00 |  |
| AMULA comp : hammer |  |  |  |  |  | 0.766 |
| - Delta | 0.11 | 0.78 | 0.00 | 0.00 | 1.00 |  |
| - Pre | 1.78 | 0.44 | 2.00 | 2.00 | 2.00 |  |
| - Post | 1.89 | 0.60 | 2.00 | 2.00 | 2.00 |  |
| AMULA comp : towel |  |  |  |  |  | 0.572 |
| - Delta | 0.22 | 0.97 | 0.00 | 0.00 | 1.00 |  |
| - Pre | 2.11 | 0.78 | 2.00 | 2.00 | 3.00 |  |
| - Post | 2.33 | 0.71 | 2.00 | 2.00 | 3.00 |  |
| AMULA comp : overhead shelf |  |  |  |  |  | 0.233 |
| - Delta | 0.33 | 0.71 | 0.00 | 0.00 | 1.00 |  |
| - Pre | 2.33 | 0.50 | 2.00 | 2.00 | 3.00 |  |
| - Post | 2.67 | 0.50 | 3.00 | 2.00 | 3.00 |  |
