# Supplementary material for: The SoftHand Pro: Functional evaluation of a novel, flexible, and robust myoelectric prosthesis
Source: PLoS One. 2018 Oct 15;13(10):e0205653. doi: 10.1371/journal.pone.0205653 (PMC6188862; doi:10.1371/journal.pone.0205653)
Supplement: S3 Table — The table below presents summary statistics for limb-intact participants before and after training with the SoftHand Pro. The p-value is from a signed rank test to test if the median change is significantly different from zero. Note: The Jebsen “writing” sub-task was not performed in limb-intact participants. (DOCX) [file pone.0205653.s003.docx]

**S3 Table. Training effect (Delta) in limb-intact participants.** The table below presents summary statistics for limb-intact participants before and after training with the SoftHand Pro. The p-value is from a signed rank test to test if the median change is significantly different from zero. Note: The Jebsen “writing” sub-task was not performed in limb-intact participants.

| **Measure** | **Mean** | **SD** | **Median** | **25th %ile** | **75th %ile** | **P-value** |
| --- | --- | --- | --- | --- | --- | --- |
| B&B : Score |  |  |  |  |  | 0.141 |
| - Delta | 2.00 | 3.28 | 2.00 | 0.00 | 4.00 |  |
| - Pre | 5.44 | 2.24 | 5.00 | 4.00 | 7.00 |  |
| - Post | 7.44 | 4.42 | 7.00 | 4.00 | 11.00 |  |
| AMULA : Score |  |  |  |  |  | 0.036 |
| - Delta | 3.26 | 2.82 | 3.53 | 0.00 | 4.71 |  |
| - Pre | 16.93 | 3.14 | 17.65 | 16.47 | 18.82 |  |
| - Post | 20.19 | 2.08 | 20.00 | 18.75 | 21.76 |  |
| Jebsen : Writing |  |  |  |  |  |  |
| - Delta | NaN |  |  |  |  |  |
| - Pre | NaN |  |  |  |  |  |
| - Post | NaN |  |  |  |  |  |
| Jebsen : Simulated page turning |  |  |  |  |  | 0.441 |
| - Delta | -6.81 | 14.15 | -1.06 | -8.00 | 3.44 |  |
| - Pre | 42.60 | 15.70 | 40.00 | 34.59 | 45.00 |  |
| - Post | 35.79 | 10.81 | 38.09 | 26.00 | 45.44 |  |
| Jebsen : Lifting small, common objects |  |  |  |  |  | 0.106 |
| - Delta | -10.19 | 15.45 | 0.00 | -12.31 | 0.00 |  |
| - Pre | 112.78 | 21.30 | 120.00 | 120.00 | 120.00 |  |
| - Post | 102.59 | 25.89 | 120.00 | 90.00 | 120.00 |  |
| Jebsen : Simulated feeding |  |  |  |  |  | 0.059 |
| - Delta | -5.16 | 7.31 | -4.00 | -7.32 | -2.00 |  |
| - Pre | 20.26 | 9.70 | 19.00 | 14.09 | 20.60 |  |
| - Post | 15.10 | 4.57 | 13.19 | 11.00 | 18.25 |  |
| Jebsen : Stacking checkers |  |  |  |  |  | 0.855 |
| - Delta | 3.81 | 35.82 | 0.00 | 0.00 | 0.00 |  |
| - Pre | 99.04 | 38.06 | 120.00 | 106.43 | 120.00 |  |
| - Post | 102.85 | 28.35 | 120.00 | 106.72 | 120.00 |  |
| Jebsen : Lifting large, light objects |  |  |  |  |  | 0.407 |
| - Delta | -2.52 | 8.09 | -1.96 | -3.00 | 1.06 |  |
| - Pre | 21.46 | 8.47 | 20.00 | 17.00 | 23.00 |  |
| - Post | 18.94 | 7.76 | 17.00 | 15.04 | 21.40 |  |
| Jebsen : Lifting large, heavy objects |  |  |  |  |  | 0.722 |
| - Delta | 2.15 | 9.76 | -1.00 | -2.29 | 7.43 |  |
| - Pre | 20.04 | 5.40 | 19.00 | 16.16 | 21.00 |  |
| - Post | 22.19 | 8.83 | 24.16 | 13.87 | 28.25 |  |
| AMULA comp : comb |  |  |  |  |  | 1.000 |
| - Delta | 0.11 | 0.33 | 0.00 | 0.00 | 0.00 |  |
| - Pre | 2.00 | 0.50 | 2.00 | 2.00 | 2.00 |  |
| - Post | 2.11 | 0.33 | 2.00 | 2.00 | 2.00 |  |
| AMULA comp : t-shirt on |  |  |  |  |  | 1.000 |
| - Delta | 0.00 | 1.00 | 0.00 | 0.00 | 1.00 |  |
| - Pre | 1.56 | 0.53 | 2.00 | 1.00 | 2.00 |  |
| - Post | 1.56 | 0.73 | 2.00 | 1.00 | 2.00 |  |
| AMULA comp : t-shirt off |  |  |  |  |  | 1.000 |
| - Delta | 0.00 | 1.12 | 0.00 | -1.00 | 1.00 |  |
| - Pre | 1.44 | 0.73 | 2.00 | 1.00 | 2.00 |  |
| - Post | 1.44 | 0.73 | 2.00 | 1.00 | 2.00 |  |
| AMULA comp : button shirt |  |  |  |  |  | 0.120 |
| - Delta | 0.56 | 0.88 | 1.00 | 0.00 | 1.00 |  |
| - Pre | 1.22 | 0.83 | 1.00 | 1.00 | 2.00 |  |
| - Post | 1.78 | 0.83 | 2.00 | 2.00 | 2.00 |  |
| AMULA comp : zipper |  |  |  |  |  | 0.588 |
| - Delta | 0.22 | 1.09 | 0.00 | -1.00 | 1.00 |  |
| - Pre | 1.44 | 0.73 | 2.00 | 1.00 | 2.00 |  |
| - Post | 1.67 | 0.50 | 2.00 | 1.00 | 2.00 |  |
| AMULA comp : sock |  |  |  |  |  | 0.424 |
| - Delta | 0.25 | 0.71 | 0.00 | 0.00 | 1.00 |  |
| - Pre | 1.56 | 0.53 | 2.00 | 1.00 | 2.00 |  |
| - Post | 1.75 | 0.71 | 2.00 | 2.00 | 2.00 |  |
| AMULA comp : shoe |  |  |  |  |  | 0.773 |
| - Delta | 0.11 | 0.60 | 0.00 | 0.00 | 0.00 |  |
| - Pre | 1.44 | 0.88 | 2.00 | 1.00 | 2.00 |  |
| - Post | 1.56 | 0.73 | 2.00 | 1.00 | 2.00 |  |
| AMULA comp : cup |  |  |  |  |  | 0.773 |
| - Delta | 0.11 | 0.60 | 0.00 | 0.00 | 0.00 |  |
| - Pre | 1.56 | 0.53 | 2.00 | 1.00 | 2.00 |  |
| - Post | 1.67 | 0.50 | 2.00 | 1.00 | 2.00 |  |
| AMULA comp : fork |  |  |  |  |  | 0.011 |
| - Delta | 0.78 | 0.44 | 1.00 | 1.00 | 1.00 |  |
| - Pre | 1.89 | 0.33 | 2.00 | 2.00 | 2.00 |  |
| - Post | 2.67 | 0.50 | 3.00 | 2.00 | 3.00 |  |
| AMULA comp : spoon |  |  |  |  |  | 0.072 |
| - Delta | 0.44 | 0.53 | 0.00 | 0.00 | 1.00 |  |
| - Pre | 1.89 | 0.33 | 2.00 | 2.00 | 2.00 |  |
| - Post | 2.33 | 0.50 | 2.00 | 2.00 | 3.00 |  |
| AMULA comp : writing |  |  |  |  |  | 0.072 |
| - Delta | 0.44 | 0.53 | 0.00 | 0.00 | 1.00 |  |
| - Pre | 1.78 | 0.44 | 2.00 | 2.00 | 2.00 |  |
| - Post | 2.22 | 0.44 | 2.00 | 2.00 | 2.00 |  |
| AMULA comp : cutting |  |  |  |  |  | 0.424 |
| - Delta | 0.22 | 0.67 | 0.00 | 0.00 | 1.00 |  |
| - Pre | 2.11 | 0.33 | 2.00 | 2.00 | 2.00 |  |
| - Post | 2.33 | 0.71 | 2.00 | 2.00 | 3.00 |  |
| AMULA comp : doorknob |  |  |  |  |  | 0.424 |
| - Delta | 0.22 | 0.67 | 0.00 | 0.00 | 1.00 |  |
| - Pre | 1.67 | 0.50 | 2.00 | 1.00 | 2.00 |  |
| - Post | 1.89 | 0.33 | 2.00 | 2.00 | 2.00 |  |
| AMULA comp : phone |  |  |  |  |  | 0.072 |
| - Delta | 0.44 | 0.53 | 0.00 | 0.00 | 1.00 |  |
| - Pre | 1.89 | 0.33 | 2.00 | 2.00 | 2.00 |  |
| - Post | 2.33 | 0.50 | 2.00 | 2.00 | 3.00 |  |
| AMULA comp : hammer |  |  |  |  |  | 0.073 |
| - Delta | 0.56 | 0.73 | 1.00 | 0.00 | 1.00 |  |
| - Pre | 1.44 | 0.73 | 2.00 | 1.00 | 2.00 |  |
| - Post | 2.00 | 0.71 | 2.00 | 2.00 | 2.00 |  |
| AMULA comp : towel |  |  |  |  |  | 0.037 |
| - Delta | 0.56 | 0.53 | 1.00 | 0.00 | 1.00 |  |
| - Pre | 2.00 | 0.00 | 2.00 | 2.00 | 2.00 |  |
| - Post | 2.56 | 0.53 | 3.00 | 2.00 | 3.00 |  |
| AMULA comp : overhead shelf |  |  |  |  |  | 0.037 |
| - Delta | 0.56 | 0.53 | 1.00 | 0.00 | 1.00 |  |
| - Pre | 1.89 | 0.33 | 2.00 | 2.00 | 2.00 |  |
| - Post | 2.44 | 0.53 | 2.00 | 2.00 | 3.00 |  |
